# Effect of ginsenosides on microbial community and enzyme activity in continuous cropping soil of ginseng

**DOI:** 10.3389/fmicb.2023.1060282

**Published:** 2023-05-05

**Authors:** Xinyue Miao, Ergang Wang, Yi Zhou, Yu Zhan, Ning Yan, Changbao Chen, Qiong Li

**Affiliations:** Jilin Ginseng Academy, Changchun University of Chinese Medicine, Changchun, China

**Keywords:** ginsenoside, microbial community, enzyme activity, environmental factors, secondary metabolites

## Abstract

Root exudates contain plant metabolites secreted by the roots into the soil, such as ginsenosides secreted by the ginseng root. However, little is known about ginseng root exudate and its impact on the chemical and microbial properties of soil. In this study, the effect of increasing concentrations of ginsenosides on the chemical and microbial properties of soil was tested. Chemical analysis and high-throughput sequencing techniques were used to evaluate the soil chemical properties and microbial characteristics following exogenous application of 0.1 mg·L^−1^, 1 mg·L^−1^, and 10 mg·L^−1^ ginsenosides. Ginsenosides application significantly altered soil enzyme activities; SOM-dominated physicochemical properties were significantly reduced which altered the composition and structure of the soil microbial community. In particular, treatment with 10 mg∙L^−1^ ginsenosides significantly increased the relative abundance of pathogenic fungi such as *Fusarium, Gibberella* and *Neocosmospora*. These findings indicate that ginsenosides in root exudates are important factors that may lead to increased deterioration of soil during ginseng cultivation and provided new research direction for the subsequent study on the mechanism of interaction between ginsenosides and soil microbial communities.

## 1. Introduction

Ginseng (*Panax ginseng* C. A. Meyer) is a perennial herb with a long planting history in the world and an important cash crop (Baeg and So, 2013). With the rapid economic development and shortage of soil resources, the intensive cultivation system characterized by continuous monoculture has become an important part of the current industry of ginseng production and is widely used in the world (Banerjee et al., 2019). Numerous studies have shown that ginseng has a strong contraindication to the soil, and continuous planting of ginseng seems to cause its roots to rust and rot due to soil sickness, which hinders the healthy growth of ginseng (Wang et al., 2016; Dong L.-L. et al., 2017; Dong L. et al., 2017; Zhang et al., 2022). Consequently, the yield and quality of P. ginseng cannot be guaranteed, causing huge economic losses and impeding the healthy and sustainable development of the ginseng industry (Tong et al., 2021). Studies to date suggest that several factors are associated with ginseng soil sickness, including deterioration of soil physicochemical properties and an imbalance in soil microbial communities (Dong L.-L. et al., 2017; Dong L. et al., 2017; Bao et al., 2020). The diversity and composition of soil microbial communities are essential to maintain soil health and quality (Garbeva et al., 2004). And, changes in microbial community diversity and composition are associated with a number of biotic or abiotic factors, such as cropping system, root secretions, and soil type (Green et al., 2008; Berg and Smalla, 2009).

More and more studies are confirming the relationship between root exudates and soil microbial communities (Fujimatsu et al., 2020). Phenolic acids are one of the important substances of root exudates and produced by various plants (Ma et al., 2015; Wang et al., 2018; Lin et al., 2019). For example, p-hydroxybenzoic acid released from roots of cucumber can promote the growth of pathogenic fungi and increase the density of inter-root bacteria and fungi (Zhou et al., 2012). Root exudates can also directly suppress pathogens or alter the composition of microbial communities. In Arabidopsis, the gene cluster responsible for the synthesis of specific metabolites (e.g., triterpenoids, esters, and coumarins) would prenyltransferase-terpene synthase (PT-TPS) be altered to promote *de novo* functionalization of genes, a result that reveals the function of metabolites in being able to regulate microbial communities (Lundberg et al., 2012; Stringlis et al., 2018; Chen et al., 2019).

Ginsenosides are the major active compounds in ginseng, and ginseng contains at least 20 different ginsenosides, accounting for more than 6% of the plant biomass (Yang et al., 2015; Zhang et al., 2021). As the main root metabolites of the *Panax L.*, ginsenosides can be released into the rhizosphere soil through root exudation, leaching or decomposition of plant residues (Yang et al., 2015). Although many studies have been conducted to investigate the pharmacological properties of ginsenosides, little is known about the ecological role of these important secondary metabolites once released from *Panax L*. into the surrounding soil (Ng, 2006). Furthermore, more than 65 of the known ginsenosides at different concentrations have been shown to play an important role in the growth of ginseng, and it has been demonstrated that many ginsenosides have a stimulating effect on the growth of important ginseng pathogens such as *Pythiumirregulare*, *Cylindrocarpon destructans*, *Phytophthora cactorum*, and *Fusarium solani* (Nicol et al., 2003; Yousef and Bernards, 2006; Li et al., 2020). However, there is limited information on the effect of different concentrations of ginsenosides on microbial communities leading to soil sickness.

In this study, we aimed to (1) investigate whether ginsenosides might be one of the main causes of soil sickness during ginseng cultivation; and (2) evaluate the effects of different concentrations of ginsenosides on the soil microbial community.

## 2. Materials and methods

### 2.1. Field experiment description and design

The soil used in this experiment was obtained from a ginseng plantation in Baixi Forestry Field, Fusong County, Jilin Province (127°01′-128°06′E, between 1°42′-42°49′N), which is located in south eastern Jilin province, upstream of the Songhua River, and has a temperate continental monsoon climate. Samples of soil were collected from the ginseng rhizosphere. The basic physicochemical properties of this dark brown loan soil were pH, 5.61; soil organic matter (OM) content, 139.51 (g∙kg^−1^); electrical conductivity (EC), 40.2 (μs·cm^−1^); fast-acting phosphorus (AP), 35.55 (mg∙kg^−1^); fast-acting potassium (AK), 659.3 (mg∙kg^−1^); and fast-acting nitrogen (AN), 95.68 (mg∙kg^−1^).

Sampling was carried out at the sampling location in 2019 using the S-shaped random multi-point mixed sampling method, taking the uppermost 0–20 cm of soil from each processing group. And, the five soil sampling points were mixed into one composite sample. This method meant that four composite soil samples were obtained from each treatment. After sample collection, soil fauna and plant residues were removed and 10 kg were taken in sterile sampling bags according to the quadrat method and brought back to the laboratory. One part of the samples was dried naturally in a ventilated room and then passed through a 2-mm mesh size sieve, and used for determining the physicochemical properties and enzymatic activity of the soil; the other part was used for microbial diversity analysis.

### 2.2. Experimental design

Sub-samples of soil (200 g) were added to 100 ml of distilled water and incubated in a constant temperature incubator at 25°C for 15 d. The three treatment groups, SP_1, SP_2, SP_3 represent the three ginsenosides (Shanghai yuanye Bio-Technology Co., Ltd) concentrations, 0.1 mg·L^−1^, 1 mg·L^−1^, and 10 mg L ^−1^, in 20 ml of solution, added to soil samples and incubated for 15 d. An untreated control group was included for each experiment. All soil samples were placed in wide mouth bottles containing test tubes with 5 ml deionized water, incubated at a constant temperature of 25°C, and the water content measured every 3 d and replenished when insufficient. In this experiment, 4 treatment groups were set up, and 4 parallel trials were conducted for each treatment group, totaling 16 test samples. On the 90th day, samples of culture were removed, stored at −80, and 3 g of fresh soil added.

### 2.3. Soil enzyme activity analysis

The pH of the soil was determined at a ratio of 1: 2 using a glass electrode pH meter (Jiang et al., 2019); EC was determined using a conductivity meter (Meng et al., 2013) and OM was determined using the potassium dichromate oxidation method (Flowers and Bremner, 1991) AP in soil was determined by leaching molybdenum antimony anti-colorimetric method (Kovar and Pierzynski, 2009); AK was determined by extraction using the acetamide method (Tardy et al., 2015), and AP was determined by phosphor molybdenim blue colorimetric method (Matula, 2010).

Important enzymes involved in soil nutrient cycling processes and microbial metabolism including catalase (S-CAT), laccase (SL), urease (S-UE), and sucrase (S-SC) were determined for each sample. Urease (S-UE) activity was determined using urea as substrate by colorimetric method with NH_3_^−^N. Laccase (SL) was determined using ABTS as substrate, incubated for 1 h, and detected at 420 nm by UV spectrophotometer (Eichlerova et al., 2012) Sucrase (S-SC) was determined using sucrose as a substrate and the glucose produced was measured colorimetrically (Shi et al., 2008). Catalase (CAT) activity was determined in the samples by adding H_2_O_2_ solution (Rodíguez-Kábana, 1982).

### 2.4. Soil DNA extraction and sequencing

Total DNA was extracted from 0.5 g of soil sample using the E.Z.N.A.® soil DNA kit (Omega Bio-Tek, Norcross, GA, United States; Wang et al., 2020) according to the manufacturer’s instructions, and DNA concentration and purity were determined using a NanoDrop2000. Each soil sample was extracted in triplicate and the three DNA solutions were thoroughly mixed. The DNA samples were stored in a container in a − 20°C refrigerator for subsequent analysis.

Primers 338F and 806R were used to amplify the V3-V4 variable region of the 16srRNA gene. Transcript amplicons were used to amplify the fungal gene spacer region using primer ITS1 (Nottingham et al., 2018). The initial denaturation steps were 5°C pre-denaturation for 3 min, 27 cycles (95°C denaturation for 30 s, 55°C annealing for 30 s, 72°C extensions for 30 s), followed by 72°C stable extensions for 10 min, and finally storage at 4°C (PCR instrument: ABI GeneAmp® model 9,700). the PCR reaction system was: 5× Trans Start Fast Pfu buffer 4 μL, 2.5 mM dNTPs 2 μL, upstream primer (5uM) 0.8 μL, downstream primer (5uM) 0.8 μL, Trans Start Fast Pfu DNA polymerase 0.4 μL, template DNA 10 ng, made up to 20 μL 3 replicates per sample.

### 2.5. Illumina Miseq sequencing and data processing

PCR products from the same sample were mixed and recovered using a 2% agarose gel, purified using the AxyPrep DNA Gel Extraction Kit (Axygen Bios-sciences, Union City, CA, United States), detected by 2% agarose gel electrophoresis, and quantified using a Quantus™ Fluorometer (Promega, United States). A fluorometer (Promega, USA) was used to quantify the recovered products. Sequencing was performed using Illumina’s Miseq PE300 platform (Shanghai Majorbio Bio-Pharm Technology Co Ltd.). The raw reads were deposited into the National Center for Biotechnology Information (NCBI) Sequence Read Archive (SRA) database (PRJNA886310).

The raw sequenced sequences were quality-controlled using faster (Nottingham et al., 2018) software, spliced using FLASH (Magoc and Salzberg, 2011) software, and sequences were OTU clustered and chimeras removed based on 97% (Goebel and Stackebrandt, 1994; Edgar, 2013) similarity using UPARSE (Edgar, 2013) software. Each sequence was annotated with species classification using an RDP classifier (Wang et al., 2007), compared to the Silva 16S rRNA database (v138), and a comparison threshold of 70% was set.

### 2.6. Statistical analysis

Experimental data were organized using Microsoft Excel. The statistical software SPSS 21.0 was used for one-way analysis of variance (ANOVA), and the value of p threshold of <0.05 was used to characterize significant differences between the three groups of data. Sequences were clustered at 97%, similarity analysis of OTUs was performed using UPARSE software, and chimeras were removed using UCHIME software. The analysis was performed according to different classification levels, richness and diversity indices. In the heat map analysis, Spearman’s rank correlation coefficients were calculated to assess the relationship between soil properties and microbial communities. Principal coordinate analysis (PCOA) was performed based on Bray-Curtis distance matrices in bacteria and fungi, respectively, to visualize pairwise community differences between samples. VIF (Variance Inflation Factor) was used to filter out the environmental factors with VIF > 10.

All graphics are built using the drawing software GraphPad Prism 8.01 and the Majorbio platform.

## 3. Result

### 3.1. Effects of different concentrations of ginsenoside on soil physicochemical properties and enzyme activities

#### 3.1.1. Soil physicochemical analysis

Results from ANOVA showed that most soil physicochemical properties (pH, EC, AN, AP, AK and OM) changed significantly following application of the different ginsenosides concentration treatments (Table 1). The overall soil pH was significantly lower than the control and soil acidity increased after the application of different concentrations of ginsenosides treatments. In addition, EC, AN, and AK contents were significantly increased by 8.5, 7.33, and 33.47% in the SP_2 treatment group compared to the untreated control (CK) (*p* < 0.05). In the SP_1 group, EC and AN were the lowest, differing from the untreated control group (CK) by 4.77, 12.93, and 13.91%, respectively (p < 0.05). There was no significant difference in AP levels between the SP_1 and SP_2 groups. However, the SP_3 group had a significantly lower AP value than the untreated control (CK) group (at 2.59%). In addition, the OM values were significantly lower in the three different concentration treatment groups compared to the untreated control (CK).

**Table 1:** Changes of soil physical and chemical properties under different concentrations of ginsenosides.

Parameter	Untreated control (CK)	SP_1 0.1 mg∙L^−1^	SP_2 1 mg∙L^−1^	SP_3 10 mg∙L^−1^
pH	5.61 ± 0.03^ab^	5.64 ± 0.06^a^	5.43 ± 0.06^c^	5.56 ± 0.11^b^
EC (us∙cm^−1^)	40.20 ± 2.42^c^	35.43 ± 4.53^d^	48.17 ± 1.26^a^	43.63 ± 0.39^b^
AN (mg∙kg^−1^)	95.68 ± 2.61^b^	82.75 ± 5.06^c^	103.01 ± 6.40^a^	95.37 ± 2.58^b^
AK (mg∙kg^−1^)	659.30 ± 14.9^b^	577.56 ± 17.42^c^	692.77 ± 8.81^a^	506.06 ± 13.41^d^
AP (mg∙kg^−1^)	35.55 ± 0.39^a^	34.40 ± 0.78^a^	35.10 ± 0.69^a^	32.96 ± 0.46^b^
OM(g∙kg^−1^)	139.51 ± 3.78^a^	125.60 ± 5.72^b^	127.39 ± 2.10^b^	128.41 ± 4.28^b^

Values in the table are “mean ± standard deviation.” Different lowercase letters in the same column indicate significant differences (*p* < 0.05).

#### 3.1.2. Soil enzyme activity analysis

Compared to the untreated control (CK), we observed significant differences with a significant decrease in CAT activity, which was directly correlated with an increase in ginsenosides concentration (Table 2). Urease activity (S-UE) increased in the SP_1 group, but with an increase in ginsenosides concentration, the S-UE activity in the SP_2 group and SP_3 group significantly decreased compared to the untreated control (CK). In contrast, the S-SL activity of the SP_2 group was significantly higher than that of the SP_1 and SP_3 groups, which was not significantly different from that of the untreated control (CK). Sucrase (S-SC) activity gradually decreased, and in the SP_1 group was close to that of the untreated control (CK). However, the activity gradually decreased with an increase in ginsenosides concentration. The four soil enzyme activities showed increased activity in each individual treatment group, but overall decreased with the increase in ginsenosides concentration.

**Table 2:** Effects of different concentrations of ginsenosides on enzyme activities in soil.

	Untreated control (CK)	SP_1 0.1 mg∙L^−1^	SP_2 1 mg∙L^−1^	SP_3 10 mg∙L^−1^
CAT(U∙g^−1^)	426.03 ± 6.22^a^	387.22 ± 16.35^b^	334.59 ± 12.50^c^	306.96 ± 4.59^d^
S-UE (U∙g^−1^)	775.80 ± 2.90^b^	796.56 ± 2.42^a^	585.10 ± 2.89^c^	400.33 ± 5.57^d^
S-SL (U∙g^−1^)	93.48 ± 1.24^a^	82.50 ± 1.44^b^	91.19 ± 1.54^a^	60.28 ± 1.37^c^
S-SC (U∙g^−1^)	23.18 ± 0.87^a^	22.95 ± 1.24^a^	10.43 ± 0.46^c^	16.99 ± 0.74^b^

CAT: catalase; SL: laccase; S-UE: urease; S-SC: sucrase.

### 3.2. Effect of different concentrations of ginsenosides on the diversity of soil bacterial communities

A total of 693,116 high-quality 16srRNA sequences were obtained from the 16 soil samples these sequences have been distributed with 97% similarity in as many different OTUs, with flat a and b dilution curves in the samples for measuring the changes in microbial communities at different concentrations of ginsenosides. This indicated that the data obtained from this study was large enough for bacterial and fungal diversity analysis.

**Figure 1:**
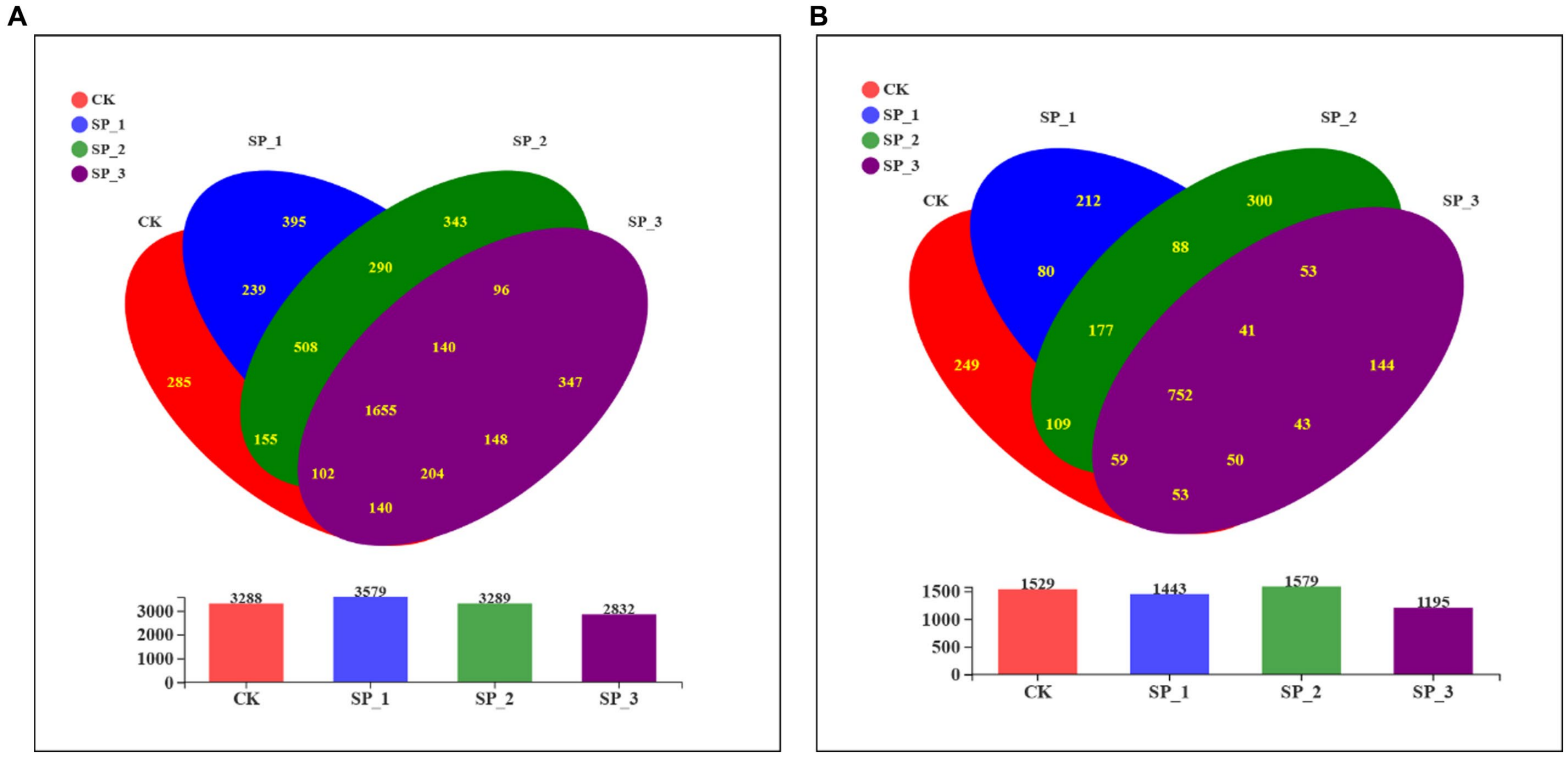
Venn diagram of microbial communities following treatment of soil with different concentrations of ginsenosides. **(A)** Bacterial community; **(B)** fungal community.

The α-diversity indices, including the number of species observed and the diversity and richness indices of bacteria and their fungal pools, were different in all soil samples (Table 3). For bacteria, the community diversity and richness (Shannon = 4.122, ace = 456.596, Chao1 = 456.335) of soils in the SP_3 group was the lowest of all samples (*p* < 0.05); for fungi, SP_1 (Shannon =4.122, ace =249.145, Chao1 = 247.547) was the lowest (p < 0.05). In particular, the diversity indices of bacteria and fungi showed similar trends. Fungal and bacterial alpha diversity indices were significantly different between the SP_3 and untreated control (CK) groups. The estimated abundance of bacteria and fungi (ACE and Chao1) reached the highest values recorded under SP_2 and SP_1, respectively. The SP_3 group resulted in a significant reduction in the number of bacterial and fungal communities OTUs under the three different treatments (Figure 1). This indicates that the species richness and diversity of bacterial and fungal communities in the soil were differentially affected in soils treated with different concentrations of ginsenosides.

**Table 3:** Microbial community under different concentrations of ginsenosides α diversity index.

	Bacteria	Fungi			
Index	Shannon	Simpson	ace	Chao1	Shannon	Simpson	ace	Chao1
Untreated control (CK)	4.413a	0.027a	510.726a	512.206ab	3.320b	0.104a	274.237a	275.701a
SP_1	4.298a	0.033a	493.174a	503.522ab	3.345b	0.111a	284.186a	293.375a
SP_2	4.427a	0.027a	508.874a	520.929a	3.218ab	0.118a	262.369a	263.535a
SP_3	4.122b	0.340a	456.596b	456.335b	2.927b	0.178b	249.145b	247.547b

Different lowercase letters indicate significant differences under different concentrations of ginsenoside treatment (*p* < 0.05), data are mean values.

### 3.3. Soil microbial community distribution

**Figure 2:**
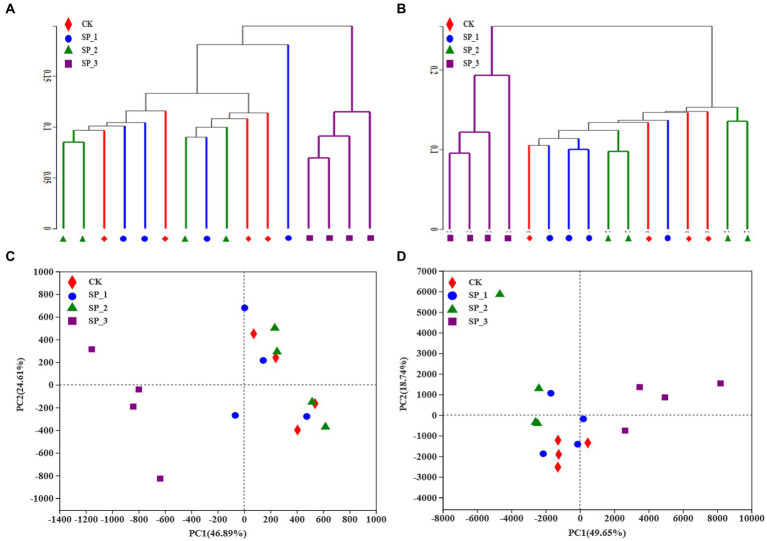
Principal component analysis of microbial communities following treatment of soil with different concentrations of ginsenosides. **(A)** Principal component analysis of bacterial community; **(B)** principal component analysis of fungal community; **(C)** hierarchical cluster analysis of bacterial samples; **(D)** hierarchical cluster analysis of fungal samples.

Principal coordinate analysis (PCoA) grouped the soils from the four groups (Figure 2C) and, analysis of similarity based on Bray-Curtis distance showed significant differences in the distribution of bacterial communities among the four groups. Interestingly, the other three groups clustered on the same side but distantly from the SP_3 group (Figure 2C). The hierarchical clustering analysis (Figure 2A) was validated. In particular, the other three groups clustered together with the SP_3 group at a clustering level of 0.1 (Figure 2B). The distribution of fungal communities in the four groups was also clearly different, with the SP_3 group being very distant from the other three groups (Figure 2D). The distribution of bacterial and fungal communities showed that the microbial populations dramatically changed after the application of various ginsenoside concentrations.

### 3.4. Soil microbial community structure

**Figure 3:**
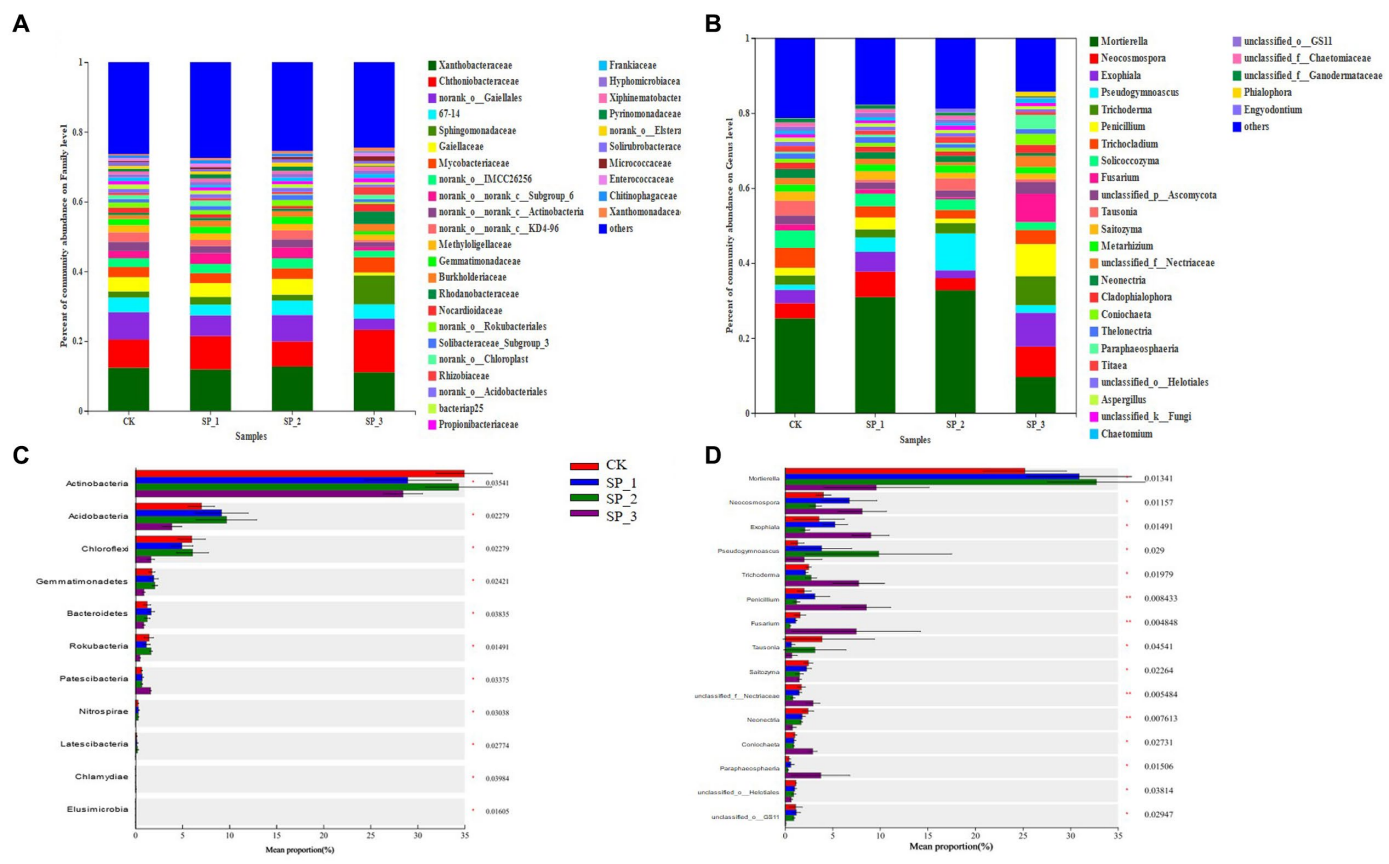
The difference in microbial community composition and relative abundance at phylum level. **(A)** Community composition at the level of bacterial family; **(B)** community composition at the level of fungi genera; **(C)** difference in relative abundance at the level of bacterial phylum; **(D)** difference in relative abundance at the level of fungal phylum (*0.01 < *p* ≤ 0.05, **0.001 < *p* ≤ 0.01, ***p ≤ 0.001).

The four groups were also studied for soil microbial community composition at different levels. The main bacterial families were *Xanthobacteraceae*, *Chthoniobacteraceae*, *Gaiellales*, *Sphingomonadaceae*, *Gaiellaceae*, *Mycobacteriace*, *Methyloligellaceae Burkholderiaceae*, *Rhodanobacteraceae*, *Nocardiodaceae*, *Nocardionidaceae*, *Rhizobiaceae*, *Propionibacteriaceae*, etc., which accounted for about 65% of the total soil bacteria (Figure 3A). In addition, some species were emphasized despite their low relative abundance, such as *Frankiaceae*, *Hyphomicrobiaceae*, *Xiphinematobacterace*, *Pyrinomonadaceae*, etc. However, the relative abundances of these clades differed significantly between the four groups, especially in the relative abundances of *Actinobacteria*, *Acidobacteria*, *Chloroflexi*, and *Gemmatimonadetes* (*p* < 0.05) (Figure 3C). Compared to, *Chthoniobacteraceae*, *Mycobacterium*, and *Rhodanobacteraceae* the relative abundance of the SP_3 group was significantly higher (Figure 3A). *Gaiellales* showed a significant decrease in relative abundance with increasing concentration of exogenously added ginsenosides. The relative abundance of *Gaiellaceae* and others in SP_2 increased significantly.

As in (Figure 3B), among the dominant fungal genera, *Mortierella*, *Neocosmopora*, *Exophiala*, *Pseudogymnoascus*, *Trichoderma*, *Penicillium*, *Trichocladium*, *Soilcoccozyma*, *Fusarium unclassified*_*p*__*Ascomycota*, *Tausonia*, etc. together accounted for about 80% of the soil. Distinct from the other treatment group. Notably the relative abundance of *Pseudogymnoascus* reached the highest in the SP_2 group. Compared with the untreated control (CK), the community structure of both fungi and bacteria changed significantly after application of ginsenosides, verifying the speculation of this study.

**Figure 4:**
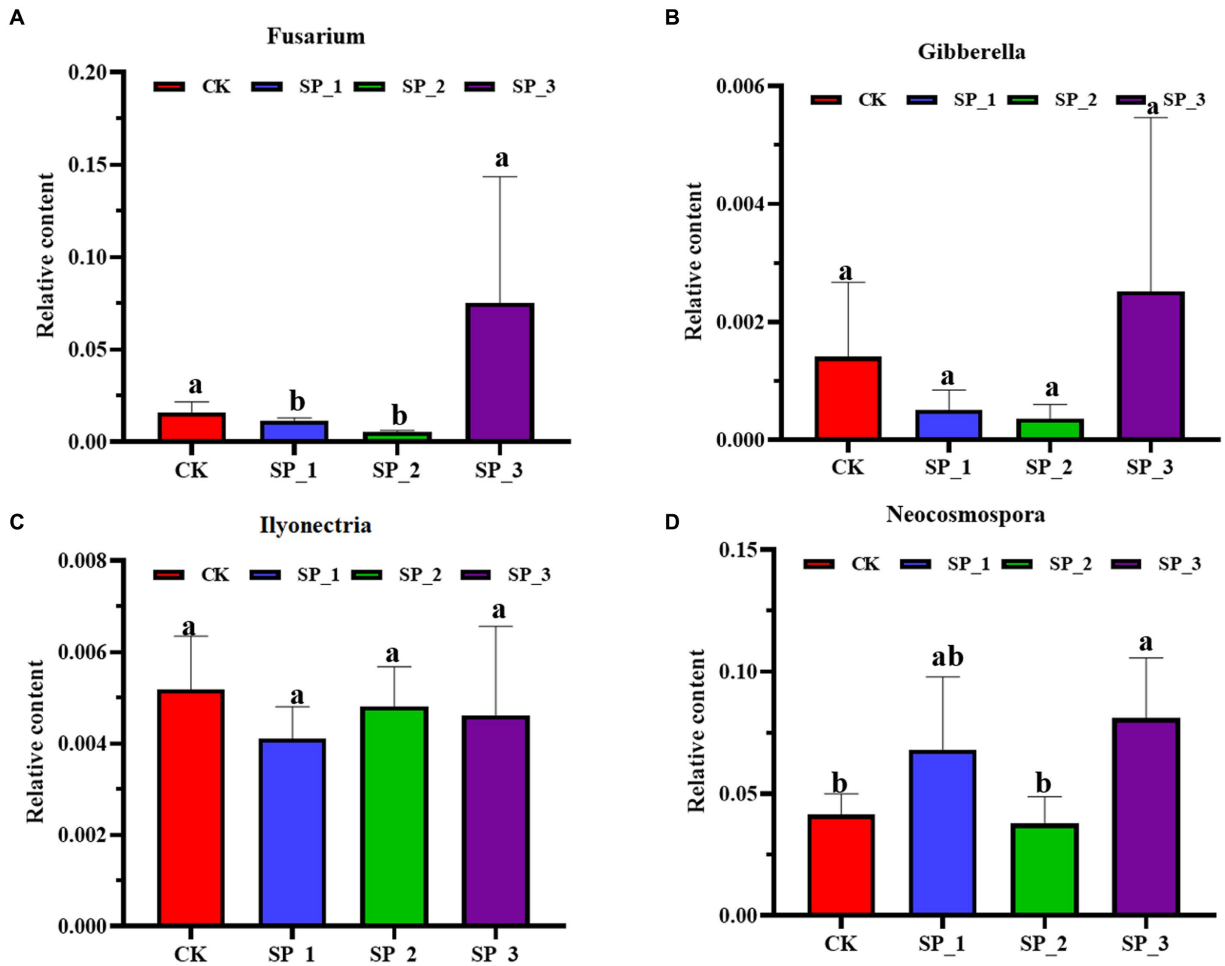
Relative abundance of pathogenic fungi under different concentrations. **(A)** *Fusarium* **(B)** *Gibberella* **(C)** *Ilyonectria* **(D)** *Neocosmospora* (relative abundance error bar represents the standard error of the average value of four repetitions). The letters indicate significant difference at *p*< 0.05 according to one-way analysis of variance (ANOVA) among treatments.

The relative abundance of the pathogenic fungi *Fusarium*, *Gibberella*, and *Neocosmospora* increased with an increase in ginsenosides concentration (Figure 4). In contrast to the untreated control (CK), the relative abundance of the three pathogenic bacteria was significantly enhanced in the SP_3 treatment group. Interestingly, the trend of relative abundance change was similar in the three groups, with SP_1 relative abundance higher than SP_2. *Ilyonectria*, unlike the other three groups of pathogenic fungi, showed less change in relative abundance after exogenous application of ginsenoside.

### 3.5. Functional prediction of soil microflora under different concentrations of ginsenoside treatment

The microbial community composition of soil samples was closely related to environmental conditions, which have been shown to be highly relevant to microbial community function. For example, microbial communities may differ significantly in similar environments, while their community functions may be similar (Gibbons, 2017). Therefore, in addition to revealing the composition and interactions of microbial communities in soil, it is particularly important to reveal differences in the metabolic functions of microbial populations in soil samples treated with different concentrations of ginsenosides. In this study, we used Tax4Fun as a reference database to explore the changes in metabolic potential in the soil after exogenously applied ginsenosides treatment at different concentrations (Aßhauer et al., 2015). This method was used by comparing metabolic pathways predicted based on genes and genomic encyclopedia (KEGG) between the treated and control samples (Aßhauer et al. 2015). Inter-root soil bacterial functions include six classes of primary metabolic pathways predicted as Cellular Processes, Environmental Information Processing, Genetic Information Processing, Human Diseases, and Metabolism The predicted results (Table 4) showed that the main pathways of KEGG predicted at level 1 in the treated and control samples were controlled by Metabolism and Environmental Information Processing (65 and 17%). Metabolism and Organismal Systems pathways were significantly different from the other treatment groups (*p* < 0.05).

**Table 4:** Relative abundance of primary metabolic functions of soil bacterial communities under different concentrations of ginsenosides.

Pathway level 1	Untreated control (CK)	SP_1	SP_2	SP_3
Cellular processes	0.0356 a	0.0371 b	0.0365 ab	0.0362 ab
Environmental information processing	0.1779 b	0.1821 c	0.1798 bc	0.1624 a
Genetic information processing	0.1050 a	0.1058 a	0.1037 a	0.1112 b
Human diseases	0.0156 a	0.0159 a	0.0156 a	0.0172 b
Metabolism	0.6540 b	0.6475 a	0.6525 b	0.6610 c
Organismal systems	0.0103 b	0.0102 a	0.0103 b	0.0105 c

Different lowercase letters indicate significant differences under different concentrations of ginsenoside treatment (*p* < 0.05), data are mean values.

**Figure 5:**
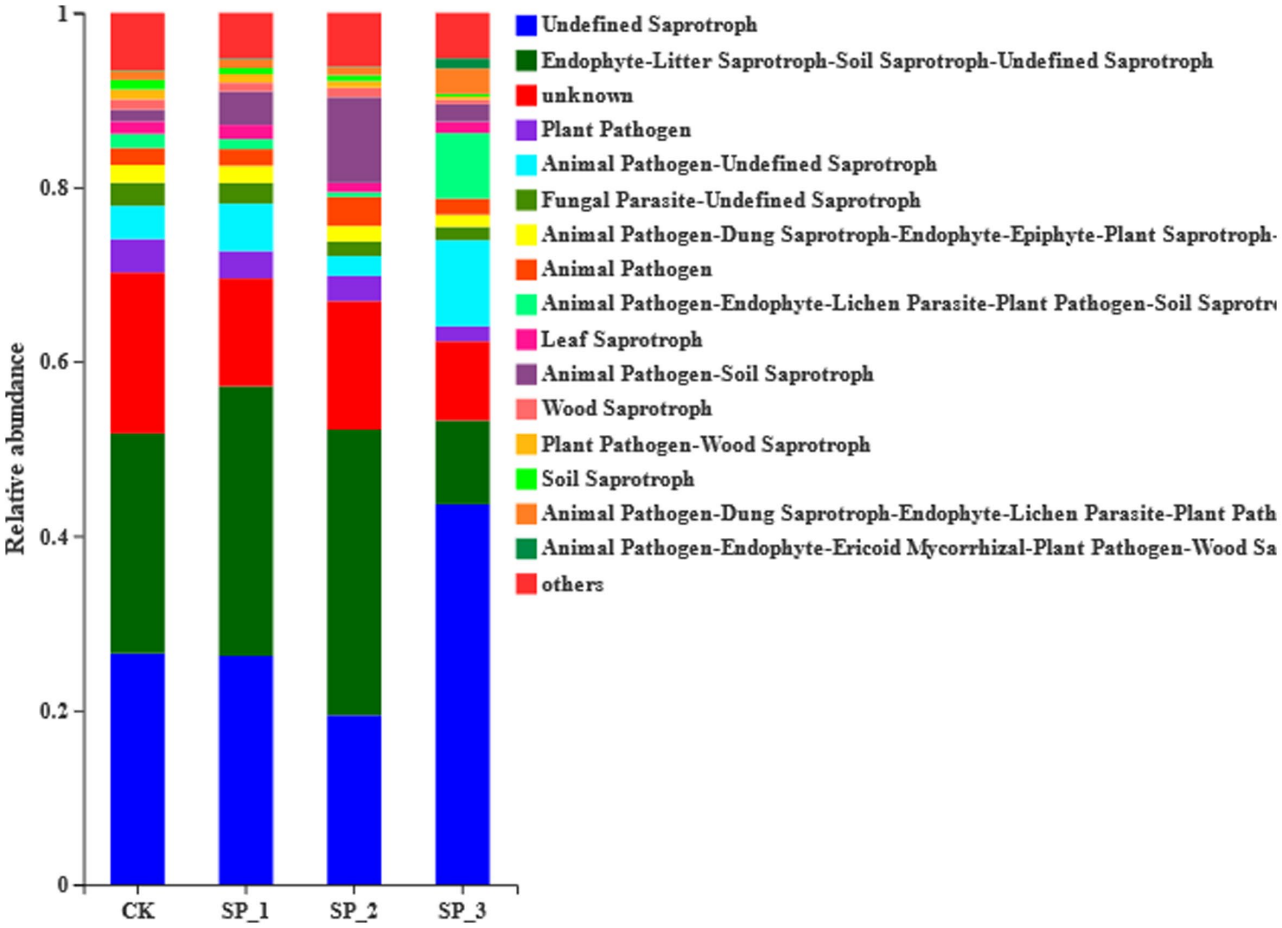
Prediction distribution of Tax4Fun gene function.

According to the results of FUNGuild functional prediction analysis (Figure 5), the fungal community trophic types included pathotrophs, saprotrophs, and symbiotrophs. The untreated control (CK) was dominated by saprotrophs with litter saprotrophs accounting for 26% of the total community. Plant-pathogens were significantly lower in the three treatment groups, and litter saprotrophs were significantly higher (7%) in the SP_2 treatment group than in the untreated control (CK) group. Animal pathogens were significantly increased in the SP_3 treatment group compared to all the other treatment groups.

### 3.6. Relationship between key soil factors, enzyme activity, and microbial structure

**Figure 6:**
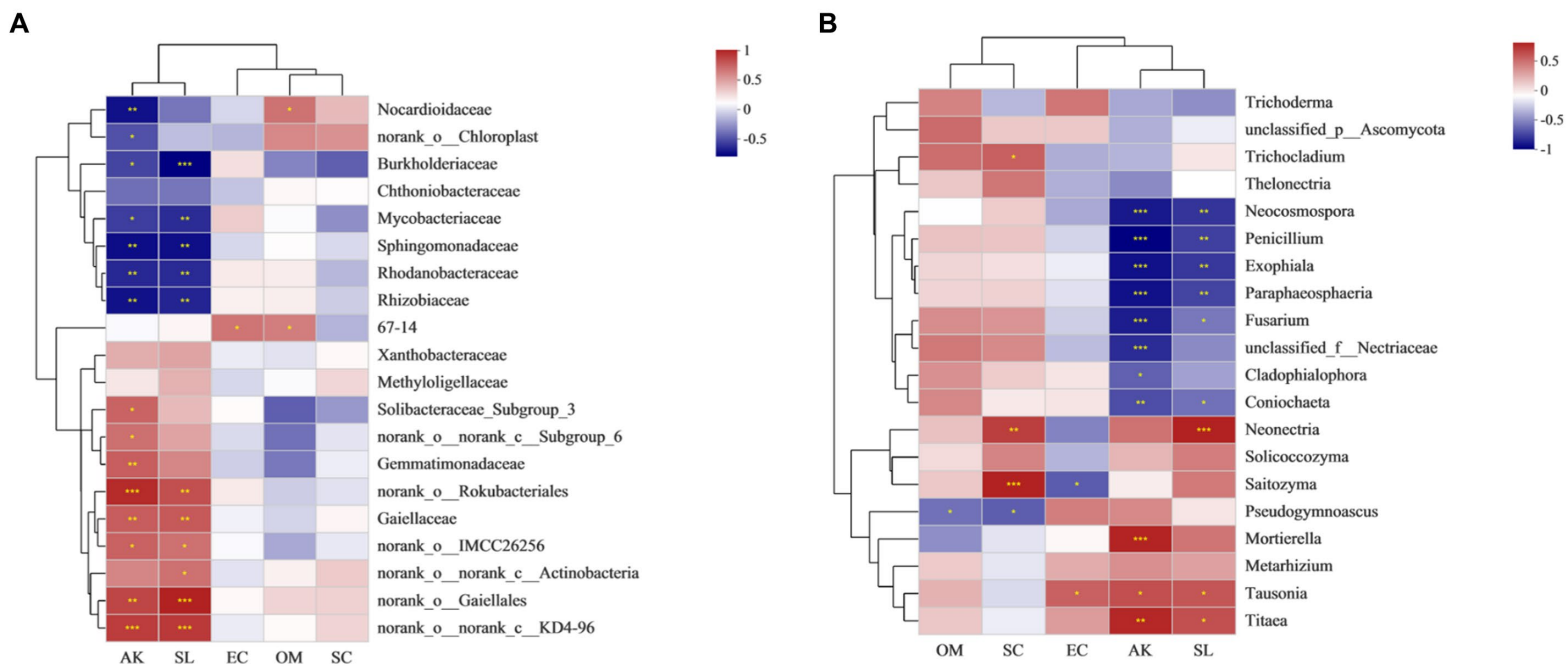
Correlation heat map of Spearman coefficient: **(A)** Heat map of Spearman correlation coefficient between soil nutrient factors, enzyme activities and rich bacterial families; **(B)**: Heat map of Spearman correlation coefficient between soil nutrient factors, enzyme activities and abundant fungal genera. (*0.01 < *p* ≤ 0.05, **0.001 < *p* ≤ 0.01, ***p ≤ 0.001).

As might be expected, environmental factors have an effect on the growth of ginseng. Five factors with low multicollinearity were screened for environmental factors (Table 5). Key soil factors such as OM, and EC showed significant correlations with increasing ginsenosides concentrations. The effects of three different concentrations of ginsenosides on soil nutrient factors and enzyme activities were investigated and showed complex relationships. Heat-map-based correlation analysis showed that *Sphingomonadaceae*, and *Mycobacteriaceae* had the highest relative abundance compared to other families and were significantly affected by AK (*p ≤ 0.05; ** *p* ≤ 0.01) (Figure 6A). *Gaiellaceae* belonging to the phylum Actinomycetes are usually present in the soil as beneficial bacteria, and showed a significant positive correlation with S-SL, and AK. Unlike the *Gaiellaceae*, *Rhizobiaceae*, and *Rhodanobacteraceae* showed a significant negative correlation with AK, and S-SL. Bacteria such as *Burkholderiaceae*, and *Sphingomonadaceae*, in contrast, showed significant negative correlations (**p ≤ 0.01; ***p ≤ 0.001) with OM and EC, while *Nocardioidaceae* showed significant positive correlations with OM. S-SL and had more significant effects on the bacterial community compared to S-SC.

**Table 5:** VIF of environmental factors.

Fungus/ Bacteria			Treatment		
	EC	OM	AK	S-SC	S-SL
VIF	1.19	1.20	1.35	1.00	1.00

VIF: variance expansion factor; EC: electrical conductivity; OM: soil organic matter; AK: acting potassium; S-SC: Sucrase; S-SL: Laccase.

Soil factors and enzymatic activity had significant effects on fungal genera, and at the genus level, the most abundant were *Mortierella*, *Neocosmospora*, and *Exophiala*. Spearman correlation coefficients were also used to assess the relationship between the top 20 fungal genera, soil factors, and enzyme activity. *Tausonia* and *Neonectria*, *Titaea* were significantly positively correlated with AK, and S-SL (**p ≤ 0.01; ***p ≤ 0.001). For *Mortierella*, the factor that significantly mimicked the abundance of this fungus was AK (*p ≤ 0.05). AK and S-SL were significantly negatively correlated with *Neocosmospora* and *Penicillium* (**p ≤ 0.01; ***p ≤ 0.001) (Table 5B). The genus was also positively correlated with EC and OM, respectively. EC was found to be a dynamic factor, positively correlated with *Saitozyma* and *Trichocladium*, and negatively correlated with *Pseudogymnoascus*. *Saitozyma* and *Exophiala* were positively correlated with S-SC and OM (Figure 6B). Pathogenic Fusarium was negatively correlated with both environmental factors and enzyme activity (*p ≤ 0.05).

## 4. Discussion

### 4.1. Effects of different concentrations of exogenous ginsenosides addition on soil chemistry and enzyme activity

Plants and microorganisms can grow in soil, and the physicochemical characteristics and enzymatic activities of soil have an impact on both the organization of microbial communities and plant growth and development (Congreves et al., 2015; Lemaire et al., 2019). An important foundation for assessing plant growth is provided by soil (AN, AP, AK), organic matter, pH, and EC, which also respond to soil fertility and health. The experiment’s fundamental hypothesis was that the soil’s physicochemical characteristics would vary depending on the ginsenoside concentration used. This hypothesis was supported by the results, which showed that the soil pH values in the SP_2 and SP_3 groups were considerably lower than those in the CK group (*p* < 0.05). This is consistent with earlier research showing that soil degradation is significantly influenced by the decline in soil pH (You et al., 2015; Gibbons, 2017; Zhang et al., 2020). It has been reported that soil pH affects the content of AN in soil (Curtin et al., 1998), also supported by our research (Table 1). Variations in soil pH following exogenous application of various ginsenoside concentrations may result in changes in soil N content, influencing the concentration of NH_4_, NO_3_^−^, and causing soil “Nitrogen” production in the soil–plant system (Klein and Logtestijn, 1994). Organic nitrogen mineralization contributes to changing soil pH by initially consuming and releasing H^+^ through nitrification during ammonification (Xu et al., 2006). The decrease in soil pH leads to changes in AN content. Phosphorus has a significant positive correlation with ginseng root weight and root diameter and is a key soil factor affecting ginseng yield and quality (Fang et al., 2019), and AP content decreased significantly with increasing ginsenoside concentration in this study. These results suggest that different concentrations of ginsenosides are one of the important factors in regulating soil physicochemical properties. Soil enzyme activities also differed compared to the changes in soil physicochemical properties in the three treatment groups. Soil sucrase, phosphatase, urease, and catalase activities were significantly reduced in all three treatment groups compared to CK (Table 2). The lowest activity of the four soil enzymes was reached in the SP_3 treatment group. It has been reported that higher soil urease activity accelerates the rate of alkaline nitrogen production and increases the content of fast-acting nitrogen (Meng et al., 2012). The decrease in urease activity in this study was speculated to be probably due to the high concentration of exogenously added ginsenoside in the SP_3 treatment group, which led to a decrease in enzyme activity due to the decrease in soil nutrient content.

### 4.2. Effect of different concentrations of exogenous ginsenosides on soil microbial community

There is growing evidence that plants can alter soil microbial communities by secreting bioactive substances into the inter-rhizosphere. Here we found that exogenous application of different concentrations of ginsenosides may lead to changes in soil microbial communities. Further findings suggest that ginsenosides can enrich or inhibit fungal and bacterial communities. The application of different concentrations of ginsenosides reduced the abundance and diversity of bacterial communities in the soil. In addition, bacterial populations also decreased gradually with increasing ginsenosides.

concentration. This indicates that ginsenosides changed the structure and composition of the soil microbial community, resulting in the proliferation of certain microbial taxa as dominant species in the soil environment, but at the same time, the metabolic types of the bacterial community tended to be homogeneous Loss of microbial diversity and abundance in soils can compromise a range of system services (Shi et al., 2018). For example, the application of different concentrations of ginsenosides decreased the levels of *Acidobacteria* and *Actinobacteria* phyla in the soil. *Acidobacteria* is an important group of soil microorganism, mostly acidophilic, suitable for proliferation in acidic environments, but with slow growth rate (Shi et al., 2018). It was found that the maximum abundance of *Acidobacteria* was found in undisturbed forest soils, and when the soil nutrient structure, changed faster-growing microorganisms would replace *Acidobacteria*, resulting in a decrease in their abundance (Ward et al., 2009). This may also be the reason for the decrease in the number of *Acidobacteria* in the SP_3 treated group, i.e. highest ginsenosides treatment. This was confirmed by successive decline in the relative abundance of *Actinobacteria* after treatment with increasing concentrations of ginsenosides. *Actinobacteria* are oligotrophic bacteria and a decrease in relative abundance in the three treatment groups may be due to a decrease in soil fast-acting nutrients after ginsenosides application.

Exogenous application of different concentrations of ginsenosides, increased soil acidity gradually, leading to an increase in abundance of acidic bacteria. However, *Xanthomonadaceae* is a group of organisms suitable for growth under weakly acidic conditions, and the number of *Xanthomonadaceae* was significantly reduced following ginsenosides treatment. *Xanthomonadaceae* consume soil nutrients and infest the xylem of plants as nitrate nitrogen gradually decreases during the growth of ginseng (Matula, 2010). Dong L.-L. et al. (2017) and Dong L. et al. (2017) showed that the *Xanthomonadaceae* population increased by 160% during three consecutive years of western ginseng cultivation. The increase in *Xanthomonadaceae* population may have led to an increase in various plant diseases (Mwangi et al., 2007). It is therefore suggested that the increase in the number of *Xanthomonadaceae* may have been due to the exogenous application of different concentrations of ginsenosides. Therefore, it is speculated that ginsenosides may have promoted the growth of root microorganisms.

A study conducted by Canadian scholars on *Panax quiquefolium* L. found that root exudate containing ginsenosides significantly promoted the growth of the soil-borne pathogens *Phytophthora cactorum* and *Pythiumirregulare*; while *Trichodermahamatum* showed a slight inhibition under the same conditions (Nicol et al., 2003). This suggests that ginsenosides can significantly affect the growth and reproduction of pathogenic microorganisms and the dynamic changes in the rhizosphere microbial community. Changes in the composition of fungal communities depend mainly on changes in the microbial community in the soil, with most microorganisms producing symbiotic or commensal associations that play a role in nutrient uptake and growth promotion, but inter-rhizosphere ginsenosides can lead to poor defence and growth of secondary soil-borne pathogens (Bednarek et al., 2010; Pascale et al., 2020). Previous studies have shown that ginsenosides stimulate the growth of soil-borne pathogens of *Panax quiquefolium* L. and *Panax notoginseng*, destroying for example *Cylindrocarpon destructans*, *Fusarium solani*, *Phytophthora cactorum,* and *Pythium irregulare* (Nicol et al., 2002, 2003). In this study, the increase in ginsenosides concentration significantly altered the core fungal group and, at the same time, the top 20 most abundant core fungal families.

Results further show that *Fusarium*, *Gibberella*, *Ilyonectria*, and *Neocosmospora* fungal families were significantly enriched in soil treated with ginsenosides. As pathogenic fungi, an increase in these organisms contributes to the increased chance of ginseng disease (Dong L.-L. et al., 2017; Dong L. et al., 2017). The interaction between Fusarium and the secondary metabolite cinnamic acid may exacerbate soil diseases (Ye et al., 2004). The increased concentration of ginsenosides promoted the growth of *Fusarium*, contributing to an increased in relative abundance. Numerous studies have shown that *Fusarium* dominates root rot causative bacteria and is the main causative agent of ginseng root rot (Ye et al., 2004). With the growth of pathogenic fungi and weak invasion causing the ginseng defence response to be stimulated, the fungal population increases, and the secondary metabolites exacerbate the occurrence of soil diseases and, the presence of the highly invasive *Ilyonectria* fungus disrupts the plant defence barrier (Wu et al., 2020). The reason for this occurrence could be the high production of hydrolytic enzymes, oxidation of phenolic compounds and sequestration of iron, Fe, by *Ilyonectria* during invasion (Rahman and Punja, 2005), and these studies are highly consistent with results of the present study. When *Ilyonectria* species are not dominant, they may also promote the growth and weak infestation of by other fungi such as *Chaetomidium*, *Candida*, *Scopulariopsis*, *Sclerotinia*, and *Penicillium*, thus aggravating soil diseases (Zhou et al., 2017). Therefore, we speculate that when the saponin concentration reaches a certain level it may be enriched with potentially pathogenic fungi.

### 4.3. Coupling environmental factors to soil microbial community structure

Soil physicochemical properties, enzyme activity, and microbial community, as important factors interact with each other to maintain the microecological environment of plant roots (Xu et al., 2021). In this study, we found that different concentrations of ginsenosides had a significant effect on the microbial diversity of ginseng rhizosphere soil, which also led to significant changes in soil physicochemical indicators and enzyme activities, in agreement with the results of previous studies (Shukla et al., 2011).

With an increase in ginsenosides concentration, soil physicochemical properties had a significant effect on the soil microbial community (Shukla et al., 2011) and EC indicating that the application of exogenous ginsenosides led to increased salinization of the soil. Increased salinization is one of the important features associated with soil quality degradation (Cambou et al., 2022). The organic matter content was significantly reduced and the sucrase activity, which is a response to the rate and content of soil organic matter conversion, was also gradually reduced. At the same time, the microorganisms that use organic matter as a carbon source to reproduce decreased, but the bacterial communities that are detrimental to ginseng growth such as *Xanthobacteraceae*, which were a negatively aorrelated with organic matter subsequently increased (Mwangi et al., 2007), leading to an increased chance of ginseng diseases. So, the decrease in organic matter content has a direct impact on changes in microbial community structure, and its composition is crucial for ginseng growth.

The correlation between microbial community and soil physicochemical indicators and enzyme activities of following application of different concentrations of ginsenosides showed that it is not only a single factor such as soil physicochemical properties, enzyme activities, or microorganisms that lead to serious soil-borne diseases of ginseng, but the result of the joint (inter) action. The increase in ginsenosides concentration directly or indirectly modulates the effect of community characteristics on soil enzyme activity, while the composition and other physicochemical properties of photosynthetic bacteria in the soil also mediate microbial growth, community structure, abundance, and diversity, and indirectly drive enzyme activity (Wang et al., 2022).

Secondary metabolites are considered to be one of the more important factors affecting soil microorganisms and driving microbial community changes at different stages of plant growth. Ginsenosides as secondary metabolites have a promoting effect on microbial communities in the soil at certain concentrations, but affect fungal communities differently when the concentration either exceeds or decreases below some critical point, but this conclusion still lacks the most direct evidence. Therefore, studying the mechanisms of secondary metabolite-microbial interactions and exploring the changes in secondary metabolite concentrations are the directions we will focus on in the future. Meanwhile, further follow-up analyses are needed to understand the changes in environmental factors affecting microbial community structure.

## 5. Conclusion

The interaction between plants and soil microorganisms mediated by the root secretion is a complex process. Soil chemistry, soil microbial community and enzyme activity showed different responses to the application of ginsenosides at increasing concentrations. This study demonstrated that alterations in ginsenosides concentrations affected soil microbial communities to some extent. Bacterial diversity in the soil decreased whereas fungal diversity increased. In addition, the community composition of soil bacteria and fungi changed, with a significant increase in the relative abundance of the pathogenic fungi *Fusarium*, *Erysipelas*, *Neospora*, and *Illinois*. Therefore, ginsenosides as secondary metabolites, may be one of the main causes of ginseng soil sickness, which provides a new research direction for the subsequent suppression of ginseng soil sickness.

## Data availability statement

The datasets presented in this study can be found in online repositories. The names of the repository/repositories and accession number (s) can be found at: https://www.ncbi.nlm.nih.gov/, SUB12118583.

## Author contributions

QL and XYM conceived the study, designed the study, and collected the data. All authors analyzed the data and were involved in writing the manuscript.

## Funding

This work was financially supported by grants from National Natural Science Foundation of China (82073969); the National Natural Science Foundation of China (82204558); the Major Science and Technology Project of Jilin Province, China (20200504003YY); the the Natural Science Foundation of Jilin Province, China (YDZJ202101ZYTS015); the Science and Technology Research Project of Education Department of Jilin Province, China (JJKH20210944KJ); and the Young Scientist Project of Changchun University of Chinese Medicine (QNKXJ2-2021ZR19). These grants were received by Professor CC and played an important role in deciding to publish and prepare manuscripts.

## Conflict of interest

The authors declare that the research was conducted in the absence of any commercial or financial relationships that could be construed as a potential conflict of interest.

## Publisher’s note

All claims expressed in this article are solely those of the authors and do not necessarily represent those of their affiliated organizations, or those of the publisher, the editors and the reviewers. Any product that may be evaluated in this article, or claim that may be made by its manufacturer, is not guaranteed or endorsed by the publisher.
