# Supplementary figures and images for: Effect of ginsenosides on microbial community and enzyme activity in continuous cropping soil of ginseng
Source: Front Microbiol. 2023 May 5;14:1060282. doi: 10.3389/fmicb.2023.1060282 (PMC10196390; doi:10.3389/fmicb.2023.1060282)

**Fig.S1.** dilution curve. a: Dilution curve of bacteria; b: Dilution curve of fungi

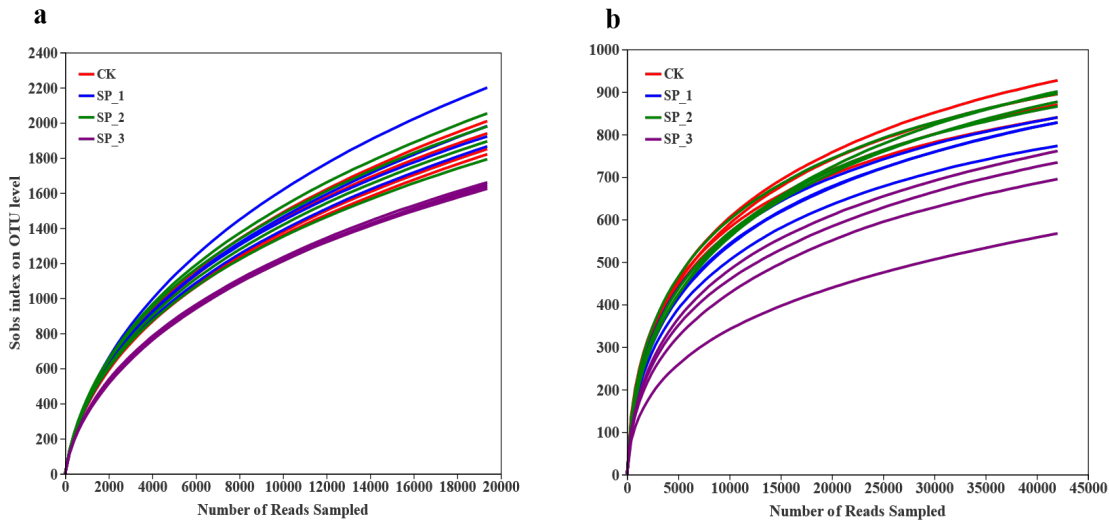

Supplement: Supplementary file 1 [file Image_1.pdf]
